# Repeated dose of prostaglandin E2 vaginal insert when the first dose fails

**DOI:** 10.4274/tjod.galenos.2021.34119

**Published:** 2021-03-12

**Authors:** Ceyda Karadağ, Sertaç Esin, Yusuf Aytaç Tohma, Ethem Serdar Yalvaç, Tuğrul Başar, Burak Karadağ

**Affiliations:** 1 Akdeniz University Faculty of Medicine, Department of Obstetrics and Gynecology, Antalya, Turkey; 2 Başkent University Faculty of Medicine, Department of Obstetrics and Gynecology, Ankara, Turkey; 3 Bozok University Faculty of Medicine, Department of Obstetrics and Gynecology, Yozgat, Turkey; 4 Ankara Gölbaşı Şehit Ahmet Özsoy State Hospital, Clinic of Obstetrics and Gynecology Ankara, Turkey; 5 Antalya Training and Research Hospital, Clinic of Obstetrics and Gynecology Antalya, Turkey

**Keywords:** Dinoprostone, labor induction, second-dose dinoprostone

## Abstract

**Objective:**

To compare the obstetric and neonatal outcomes of patients treated with repeated-dose prostaglandin E2 (dinoprostone) vaginal insert when the first dose fails.

**Materials and Methods:**

This retrospective study included 1.043 pregnant women who received dinoprostone for labor induction between November 2012 and August 2015. Pregnant women were divided into two groups according to the number of dinoprostone administrations: group 1, single-dose dinoprostone (n=1.000), and group 2, repeated-dose dinoprostone (n=43). Intrapartum, postpartum, and neonatal outcomes of the pregnant women were compared.

**Results:**

Vaginal delivery rate was 65% in group 1 and 30.2% in group 2 (p=0.001). The need for the neonatal intensive care unit was found in 44 pregnant women (4.4%) in group 1 and 6 pregnant women (13.6%) in group 2 (p=0.006).

**Conclusion:**

When obstetric and neonatal data were evaluated in our study, we observed that dinoprostone administration was associated with increased cesarean rates and adverse neonatal outcomes with repeated-dose dinoprostone when the first dose failed.

**PRECIS:** Dinoprostone administration was associated with increased cesarean rates and adverse neonatal outcomes in repeated dose of dinoprostone when first dose fails.

## Introduction

Labor induction is widely used for various maternal and fetal indications and is likely to be effective when the cervix is favorable. However, when the cervix is unfavorable (e.g. low Bishop score), iatrogenic cervical ripening is usually employed to increase the probability of vaginal delivery. For this purpose, there are two major options, which include the application of cervical ripening agents such as prostaglandins (PG), and the insertion of mechanical dilators such as cervical ripening balloons^(1,2)^. PGs efface the cervix by increasing the water content and dissolving collagen bundles^(3,4)^. In addition to these processes, myometrial contraction occurs, an advantage of PG over the use of mechanical dilatators, and collectively, these lead to cervical ripening. The efficacy of PGs for cervical ripening and labor induction has been established by randomized trials and recently by a Cochrane review^(5)^.

Dinoprostone is a prostaglandin E2 (PGE2) analogue and is approved for cervical ripening by the United States Food and Drug Administration. Endocervical gel and vaginal insert forms of dinoprostone are available^(6)^. The vaginal insert form is approved for use up to 12 and 24 hours in the United States and Europe, respectively. If labor does not ensue, or the expected Bishop change does not occur after administering PG (i.e. the cervix is still unfavorable), there is no consensus as to the preferred methods of labor induction. As a result, there are options such as repeating the PG dose, switching to mechanical dilators, oxytocin induction, or cesarean delivery^(7)^.

There are insufficient data in the literature regarding the repeated dose of dinoprostone and its safety. Our hypothesis was that repeated PGE2 administration would be associated with poor maternal and fetal outcomes. We aimed to compare the obstetric and neonatal outcomes of pregnant women treated with repeated doses of dinoprostone when the first dose fails.

## Materials and Methods

This retrospective study included 1.043 pregnant women treated with dinoprostone for labor induction at Etlik Zübeyde Hanım Women’s Health Training and Research Hospital between November 2012 and August 2015. The protocol used in this study was approved by the Institutional Review Board of Etlik Zübeyde Hanım Women’s Health Training and Research Hospital and performed in accordance with the ethical standards established by the 1964 Declaration of Helsinki.

Pregnant women with singleton pregnancies, vertex presentation, a Bishop score ≤6, and normal fetal heart rate tracing were retrospectively reviewed. A previous history of uterine surgery (e.g. cesarean section, myomectomy, septum resection), known fetal anomalies, fetal malpresentation, diagnosis with placenta previa/vasa previa, PG allergy, asthma, abnormal fetal monitorization finding, vaginal delivery contraindication and estimated fetal weight of 4.000 g or more in the ultrasonography were excluded from the study. A vaginal insert (Propess®, Ferring, Saint-Prex, Switzerland) with 10 mg slow-release dinoprostone was inserted high in the posterior vaginal fornix for cervical ripening. We recorded the insertion and retrieval times of the dinoprostone vaginal insert. After 24 hours, pregnant women with a Bishop score less than 6 were considered as non-responsive to dinoprostone. Dinoprostone was administered to pregnant women with non-responsive to dinoprostone after 24 hours. Continuous fetal monitoring was performed from the onset of the vaginal insert. Fetal heart rate classification and management were defined according to the American College of Obstetricians and Gynecologists guidelines^(8)^. Cervical opening ≥3 cm and active uterine contractions were considered active labor. Dinoprostone was retrieved in cases of active labor, uterine tetanus/tachysystole (more than five contractions in a 10-minute interval), or the presence of abnormal fetal heart monitorization. Pregnant women were divided into two groups according to the number of dinoprostone administrations: group 1 had a single dose of dinoprostone, and group 2 had a repeated dose. Intrapartum, postpartum, and neonatal outcomes of the groups were compared by reviewing patient files.

### Outcome Measures

The primary outcome was vaginal and cesarean section rates. Other outcomes considered were the interval from the start of induction to active labor and delivery, the length of the first stage of labor and the total length of labor, the need for oxytocin augmentation, the occurrence of hyperstimulation, postpartum hemorrhage, and neonatal outcomes.

### Statistical Analysis

Patient information was recorded in the Statistical Package for the Social Sciences (SPSS) Version 17.0 program. The distribution of the variables was analyzed using the Kolmogorov-Smirnov test. Continuous variables are expressed as mean ± standard deviation median (range), and categorical variables are expressed as percentages and frequencies. The independent sample t-test was used for comparisons of parametric variables, the Mann-Whitney U for non-parametric variables, and the chi-square test and Fisher’s exact tests for intermittent variables. A value of p<0.05 was considered statistically significant.

## Results

Our study included 1.064 pregnant women who underwent dinoprostone induction for labor in the perinatology unit of our hospital between November 2012 and August 2015. Twenty-one pregnant women did not meet the criteria and were excluded from the study. The remaining 1.043 pregnant women were divided into two groups as group 1 (n=1.000), which received only one dose of dinoprostone, and group 2 (n=43), which received a repeated dose. The characteristics of the groups are summarized in Table 1. Age, body mass index (BMI), mean gestational week, number of pregnancies, and amniotic fluid measurements were similar between the two groups (p>0.05). The most frequent indication for dinoprostone in both groups was post-term pregnancy. The Bishop scores during the first dinoprostone application were similar between the groups (p=0.878), whereas the Bishop score after the first application of dinoprostone was 6 (0-10) in group 1, and 1 (0-4) in group 2 (p=0.001). There was no difference in the need for oxytocin augmentation after dinoprostone retrieval in both groups (p=0.669) (Table 1). Also, 650 (65%) pregnant women in group 1 and 13 (30.2%) in group 2 had vaginal deliveries (p=0.001).

The most frequent cesarean indication was non-reassuring fetal status in group 1, and failed induction in group 2. The second stage of delivery time was similar between the two groups (p>0.05). However, latent phase and active phase durations were longer in group 2 (p=0.001, p=0.033, respectively). Ten (1%) women in group 1 and two (4.5%) in group 2 needed postpartum blood transfusions (p=0.031) (Table 2). When neonatal outcomes were evaluated, the mean APGAR score at 1 minute was 8.8±0.5 in group 1 and 8.6±0.7 (p=0.023) in group 2. Furthermore, need for neonatal intensive care unit (NICU) treatment was found in 44 (4.4%) women in group 1 and six (13.6%) women in group 2 (Table 2).

In our study, only one patient in group 1 developed a severe complication (uterine rupture). The cause of dinoprostone retrieval in this patient was active delivery and her uterus ruptured at the second stage of delivery.

## Discussion

In this study, we compared the obstetric and neonatal outcomes of pregnant women who underwent labor induction once or twice with dinoprostone. To the best of our knowledge, there is no much information in the literature about the repeated administration of dinoprostone and there are scant data on reliability and efficacy^(7,9,10)^. Our center is a tertiary teaching hospital and a reference center that has more than 16.000 deliveries annually. Additionally, our hospital is one of the largest centers of the induction of labor in Turkey. The most important finding of this study is the high cesarean rate in repeated dinoprostone administrations for women non-responsive to dinoprostone. Therefore, pregnant women should be informed that the process may result in a cesarean delivery before the second administration and other labor induction methods such as mechanical dilators and oxytocin could be offered.

In our study, most of the pregnant women were nulliparous and the most frequent indication for labor induction was post-maturity. This can be explained by the fact that post-maturity is higher in nulliparous women. On the other hand, the gestational week of women who received dinoprostone for the second time was lower than those given it once, although it was not statistically significant. Probably, the effectiveness of the dinoprostone increases as the gestational week progresses, but more patients are needed to confirm this.

The use of labor induction especially in elective delivery has increased significantly in recent years^(11)^. Dinoprostone is one of the most commonly used pharmacologic methods for the induction of labor^(12)^. Cesarean delivery, mechanical dilatation, oxytocin induction, and repeated administration of dinoprostone are alternative methods for non-responsive patients to dinoprostone during labor induction. We use repeated dinoprostone for pregnant women who are non-responsive to single-dose dinoprostone in our center.

The success of labor induction depends on many factors such as the general characteristics of the population, age, gestational week, BMI, parity, estimated fetal weight, Bishop score, and labor induction method used^(13,14,15)^. In our study, the age, gestational week, BMI, parities of pregnant women were similar between the two groups. Antonazzo et al.^(7)^ compared the results of patients who received repeated dinoprostone administrations and oxytocin induction in pregnant women who did not respond to dinoprostone. They reported that the cesarean section rate was 44.7% in 47 pregnant women who received repeated dinoprostone administration and 66% in 47 pregnant women who had oxytocin induction. In the literature, the average cesarean delivery rate for labor induction with dinoprostone is 25% (range, 10.5-38.6%), and the vaginal delivery rate within 24 hours is 59.4% (range, 38.2-81.1%)^(16,17,18,19)^. In our study, we observed that the cesarean section rate was 70.5% in 43 pregnant women who received repeated dinoprostone administrations. We speculated that this rate was extremely high, therefore patient selection for repeating dinoprostone should be performed more carefully before the second application.

Additionally, Antonazzo et al.^(7)^ found no differences in terms of neonatal outcomes (5-minute APGAR score, umbilical artery pH) between the repeated dinoprostone group and the oxytocin group. In our study, the need for NICU treatment and the need for blood transfusion were higher in group 2. Despite of literature^(7)^, our data showed that repeated administration of dinoprostone is not a safe therapeutic option when first dose fails.

### Study Limitations

The present study has some limitations such as the relatively small population for repeated dinoprostone group, the absence of other labor induction method groups for patients non-responsive to dinoprostone in labor induction, and its retrospective design. Additional well-designed randomized controlled studies are required to improve our understanding of the efficacy and outcomes (neonatal and maternal) of repeated-dose dinoprostone for women non-responsive to dinoprostone in labor induction.

## Conclusion

When we evaluated all these obstetric and neonatal data in our study, we observed that dinoprostone administration was associated with an increased rate of caesarean and adverse neonatal outcomes with the second dose compared with single-dose dinoprostone in unresponsive women. As an alternative to applying dinoprostone for the second time, mechanical or other pharmacologic methods should be tried.

## Figures and Tables

**Table 1:**
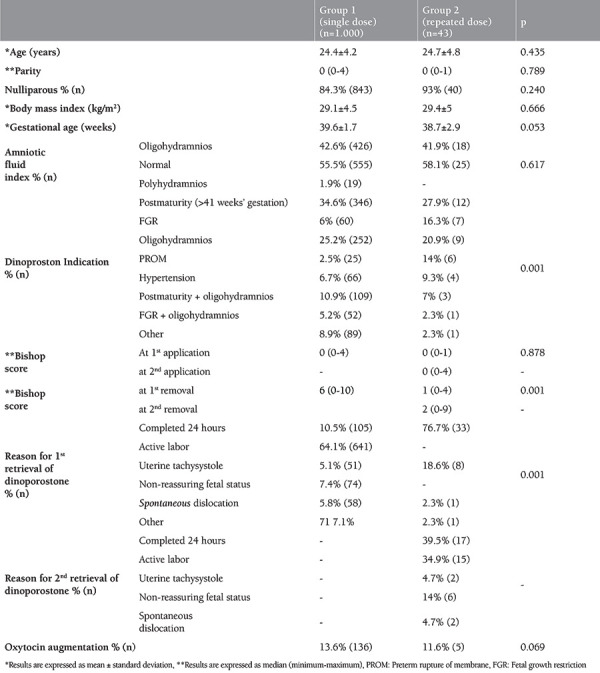
Characteristics and pre and post-Bishop scores of groups and reasons for retrieval of dinoprostone

**Table 2:**
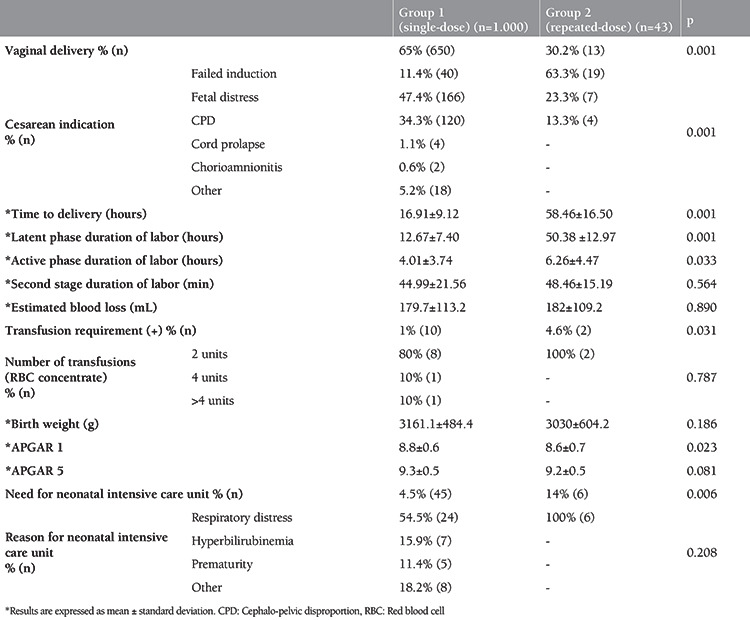
Obstetric and neonatal outcomes of groups
